# Prevalence of Pulmonary Tuberculosis - A Baseline Survey In Central India

**DOI:** 10.1371/journal.pone.0043225

**Published:** 2012-08-29

**Authors:** Vikas G. Rao, Jyothi Bhat, Rajiv Yadav, Gopi Punnathanathu Gopalan, Selvakumar Nagamiah, Manoj Kumar Bhondeley, Sharada M. Anjinappa, Jitendra Ramchandra, Vineet K. Chadha, Fraser Wares

**Affiliations:** 1 Regional Medical Research Centre for Tribals, Indian Council of Medical Research, Jabalpur, Madhya Pradesh, India; 2 National Institute for Research in Tuberculosis, Indian Council of Medical Research, Chennai, Tamilnadu, India; 3 National Tuberculosis Institute, Bangalore, Karnataka, India; 4 Stop TB Department, World Health Organization, Geneva, Switzerland; University of Otago, New Zealand

## Abstract

**Background:**

The present study provides an estimate of the prevalence of bacteriologially positive pulmonary tuberculosis in Jabalpur, a district in central India.

**Methodology/Principal Findings:**

A community based cross-sectional survey was undertaken in Jabalpur District of the central Indian state of Madhya Pradesh. A stratified cluster sampling design was adopted to select the sample. All eligible individuals were questioned for pulmonary symptoms suggestive of TB disease. Two sputum samples were collected from all eligible individuals and were examined by Ziehl-Neelsen smear microscopy and solid media culture methods. Of the 99,918 individuals eligible for screening, 95,071 (95.1%) individuals were screened. Of these, 7,916 (8.3%) were found to have symptoms and sputum was collected from 7,533 (95.2%) individuals. Overall prevalence of bacteriologically positive PTB was found to be 255.3 per 100,000 population (95% C.I: 195.3–315.4). Prevalence was significantly higher (p<0.001) amongst males (355.8; 95% C.I: 304.4–413.4) compared with females (109.0; 95% C.I: 81.2–143.3). Prevalence was also significantly higher in rural areas (348.9; 95% C.I: 292.6–412.8) as compared to the urban (153.9; 95% C.I: 123.2–190.1).

**Conclusions/Significance:**

The TB situation in Jabalpur district, central India, is observed to be comparable to the TB situation at the national level (255.3 versus 249). There is however, a need to maintain and further strengthen TB control measures on a sustained and long term basis in the area to have a significant impact on the disease prevalence in the community.

## Introduction

Tuberculosis (TB) continues to be a major public health problem in India, with an estimated 2.3 million new cases annually, making it the highest TB burden country in the world. In 2010, India alone accounted for an estimated one quarter (26%) of all TB cases worldwide [1]. The prevalence of TB disease is an important epidemiological index to measure the burden in a community and if measured periodically, will enable trends in disease prevalence to be observed over time. Epidemiological information on TB is also vital for the planning of control strategies and service delivery systems. A nation-wide disease survey conducted by the Indian Council of Medical Research (ICMR) during 1955–58 provided, for the first time, information on the TB disease situation in the general population of the country [2]. The findings revealed that the prevalence of PTB was about 400 per 100,000 population and an estimated 1.5 million infectious cases were present in the community. In view of these findings, the Government of India (GoI) implemented the National TB Programme (NTP) in 1962. Following a review of the NTP in 1992 by the GoI, the World Health Organization (WHO) and the Swedish Development Agency, the GoI resolved to intensify it's efforts to control TB and established in 1993 the Revised National Tuberculosis Control Programme (RNTCP) - an adaptation of the internationally recommended Directly Observed Treatment, Short course (“DOTS”) strategy [3]. Initially the RNTCP was implemented in a limited number of pilot areas, with large-scale implementation beginning in late 1998 in a phased manner and country-wide implementation was achieved in early 2006. There is however, limited community based data available on tuberculosis situation in different parts of the country.

In this context, the GoI decided to undertake population-based surveys to estimate the prevalence of TB disease in different parts of the country. The present study reported on is one amongst these studies approved by the GoI to provide a baseline measurement of TB prevalence in central India, and was undertaken in Jabalpur district of the central Indian state of Madhya Pradesh.

## Materials and Methods

### Study area

This cross sectional study was conducted in the urban and rural populations of Jabalpur district from January 2009 to January 2010. There are four taluqs (sub-divisions) and 1386 villages in the district. The total area of the district is 10,160 Km^2^, with a population of over 2.1 million (2001 census). The proportion of rural and urban population in the district is 43% and 57% respectively.

### Sample size

The required sample size was estimated to be about 90,000 adults aged ≥15 years for an assumed prevalence of 240/100,000 bacteriologically positive PTB cases, with a precision of 20% at 95% confidence level, a design effect of 2, and coverage for examination of at least 90%. The calculation is based on the prevalence of 400/100000 population and the assumption that 60% of the cases will be picked up by symptom elicitation [4], [5].

### Sampling design and procedure

The district was considered the sampling universe for the study. The clusters were selected using 2001 census of India population. The census population was projected for the year of survey using a population growth of 2.3% per year. A stratified cluster sampling design was adopted to select the sample from this projected population. Rural and urban clusters were sampled separately so that the rural-urban distribution in the sample was similar to the rural-urban distribution of the overall population in the district. The sample was distributed in all the taluqs and urban area using probability proportional to the size of each taluq and urban area in the district and a random sample of villages/urban wards was selected from each taluq and urban area to cover the required population.

### Census and registration

Planning visits were made to the area prior to the survey by the team leaders to inform the local leaders regarding the purpose of the survey. Group meetings were also conducted to explain the purpose of the study to the community. A house-to-house census was carried out and all permanent residents (stay ≥6 months) in each household were registered. Non-residents and temporary visitors present in the households at the time of the census were not registered. Demographic (age, sex) and other relevant data of each permanent resident was collected on pre-designed and standardized individual card in a pre-coded form by investigators trained in survey methodology by the faculty from the National Institute for Research in Tuberculosis (NIRT), Chennai (formerly known as the “Tuberculosis Research Centre”). Informed written consent was obtained from all individuals included in the survey.

### Symptom screening and sputum collection

All individuals aged 15 years and above were questioned for chest symptoms relating to TB, namely: persistent cough for two weeks or more; chest pain for one month or more; fever for one month or more; and haemoptysis anytime in last 6 months. Persons with any of these symptoms (deemed as “chest symptomatics”), and also those with a previous history of anti-TB treatment, were considered eligible for sputum collection. Two sputum samples - one spot and one overnight - were collected from each eligible individual in sterilized McCartney's bottles. Quality check for symptom elicitation was inbuilt in the survey methodology. A 10% random sample of subjects screened for symptoms by the field worker were selected independently by a supervisor and the extent of agreement was ascertained for any corrective measures.

### Processing of sputum specimens

Samples were brought to the Regional Medical Research Centre for Tribals (RMRCT) laboratory in a cold box on the same day and were kept at +4°C until processing was done. Direct smears were made from all sputum specimens. The smears were stained by Ziehl-Neelsen method and examined by trained technical staff for acid fast bacilli [6]. In addition, all positive smears and 20% random sample of negatives were read once again for quality check. All the specimens were also processed for culture by modified Petroff's method, inoculated on Lowensten-Jensen medium and were examined for growth of *M. tuberculosis* once a week for up to 8 weeks. Niacin test and growth on para-nitrobenzoic acid was done to confirm the speciation i.e. growth of *M. tuberculosis*. The quality of the procedures at the RMRCT laboratory is monitored regularly by NIRT, and the performance has been observed to be consistently satisfactory.

### Case definition

A PTB case was defined as an individual in whom any of the two sputa specimen was positive for acid fast bacilli by ZN microscopy and/or growth of *M.tuberculosis* by culture examination.

### Treatment

All bacteriologically positive cases were referred to the concerned health authorities for anti-TB treatment under the RNTCP using its standardized treatment regimens.

### Data management

All the completed cards and laboratory reports were scrutinized, checked and computerized by trained data entry operators at RMRCT. Missing value imputation was undertaken using logistic regression for symptom status of eligible individuals who could not be screened for symptoms, and for sputum smear and culture results of those individuals from whom sputum could not be collected despite having symptoms. The data was analyzed using SPSS package (13.0 version) and Stata V.11. The Chi-square test of significance was applied to the difference in proportions of symptomatic individuals and cases among different classifications. A p-value of <0.05 was considered to be statistically significant.

The study was approved by the Ethics Committee of RMRCT, Indian Council of Medical Research (ICMR), Jabalpur.

## Results

### Coverage

From January 2009 to January 2010, of 99,918 individuals eligible for screening for symptoms, 95,071 (95.1%) were screened (Table 1). The coverage was equally high (>90%) among males and females in all age groups, and in rural as well as urban areas. Of those screened, 7,916 (8.3%) individuals were found to be symptomatic. Sputum was collected from 7,533 (95.2%) of the individuals eligible for sputum examination. Coverage for symptom elicitation and sputum collection were both above 90% [Table 1].

**Table 1:** Sex wise prevalence of bacteriologically positive pulmonary tuberculosis disease.

Sex	Individuals Eligible for screening	Number Examined (%)	Eligible for sputum (%)	Number Examined (%)	Smear and/or culture positive individuals	Prevalence per 100,000 (95% C.I)
**Males**	51,499	48,105 (93.4)	4,569 (9.5)	4,239 (92.8)	170	355.8 (304.4–413.4)
**Females**	48,419	46,966 (97.0)	3,347 (7.1)	3,165 (94.6)	51	109.0 (81.2–143.3)
**Total**	99,918	95,071 (95.1)	7,916 (8.3)	7,404 (93.5)	221	255.3 (195.3–315.4)

### Symptoms

Cough was the commonest symptom (6.3%) in the screened population. This was followed by chest pain (4.3%), fever (2.0%) history of past treatment (1.1%) and haemoptysis (0.7%). Similar trends were observed in urban and rural areas, and in both sexes.

The proportion of symptommatic individuals was found to be higher among males (9.5%) compared with females (7.1%), and increased from 3.3% in the 15–24 year age group to 19.1% in the 65+ year age group with the increase in trend statistically significant (p<0.001) [Tables 1 & 2].

**Table 2:** Age wise prevalence of bacteriologically positive pulmonary tuberculosis disease.

Age group (Years)	Individuals Eligible for screening	Number Examined (%)	Eligible for sputum (%)	Number Examined (%)	Smear and/or culture positive individuals	Prevalence per 100,000 (95% C.I)
**15–24**	30,374	29,747	979 (3.3)	903	25	84.2 (54.5–124.4)
**25–34**	23,680	22,782	1,478 (6.5)	1,359	36	158.9 (111.3–219.8)
**35–44**	18,928	17,765	1,806 (10.2)	1,686	57	323.0 (244.7–418.3)
**45–54**	12,444	11,424	1,412 (12.4)	1,333	39	343.7 (244.5–469.6)
**55–64**	8,173	7,517	1,127 (15.0)	1,067	33	442.5 (304.8–620.9)
**65+**	6,319	5,836	1,114 (19.1)	1,056	31	536.5 (364.8–760.7)
**Total**	99,918	95,071	7,916 (8.3)	7,404	221	255.3 (195.3–315.4)

### Prevalence of bacteriologically positive pulmonary tuberculosis

**Figure 1:**
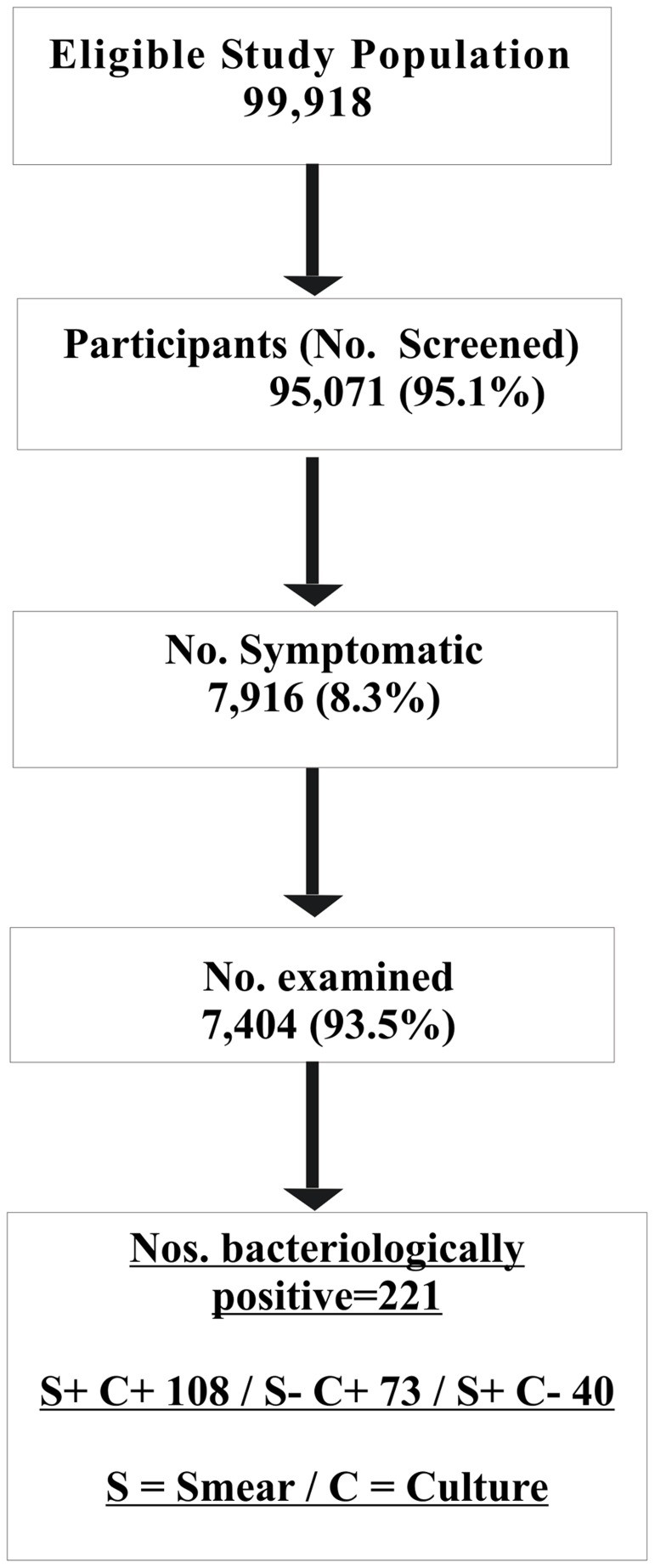
Flow chart showing study population, coverage and results.

A total of 221 individuals were found to be bacteriologically positive either by smear and/or culture [Tables 1 & 2, Figure 1]. Overall prevalence of bacteriologically positive PTB was found to be 255.3 per 100,000 population (95% C.I: 195.3–315.4). The prevalence was significantly higher (p<0.001) amongst males (355.8; 95% C.I: 304.4–413.4) compared to females (109.0; 95% C.I: 81.2–143.3) [Table 1]. Males contributed 76.9% of the total bacteriologically positive PTB cases found by the survey. Prevalence increased with age from 84.2 (95% CI: 54.5–124.4) in the 15–24 year age group to 536.5 (95% CI: 364.8–760.7) in the 65+ year age group [Table 2]. The increase in trend with age was statistically significant (p<0.001). The highest proportion of the total number of bacteriologically positive PTB cases (25.8%) was seen in the 35–44 year age group followed by 17.7% and 16.3% in the 45–54 and 25–34 year age group respectively.

Of the 221 cases detected, 108 were positive by both microscopy and culture (S+ C+), while 73 were positive by culture alone (S− C+) [Table 3]. Forty cases were positive by smear, but negative on culture (S+ C−). Of these 40 cases, 14 had a history of previous anti-TB treatment, 4 had contaminated cultures, and 10 were reported as scanty i.e. paucibacillary, smear positives.

**Table 3:** Distribution of cases by Smear and Culture results.

Sputum Result	Male (%)	Female (%)	Total (%)
**Smear −ve Culture +ve**	55 (32.4)	18 (35.3)	73 (33.0)
**Smear+ve Culture −ve**	29 (17.1)	11(21.6)	40 (18.1)
**Smear +ve Culture +ve**	86 (50.6)	22 (43.1)	108 (48.9)
**Total**	170 (100.0)	51(100.0)	221(100.0)

The prevalence of smear positive PTB cases, irrespective of culture result, was 171.9 (95% C.I: 123.7–220.1). The prevalence was significantly higher in the rural areas (348.9; 95% C.I: 292.6–412.8) as compared to that in the urban areas (153.9; 95% C.I: 123.2–190.1).

### History of current anti TB treatment

Of the total 221 bacteriologically positive PTB cases detected by the survey, 73 (33.0%) were found to be on anti TB treatment.

## Discussion

Tuberculosis remains highly prevalent in India and continues to be a leading cause of death. In view of this, there is a perceived need for developing an acceptable estimate of the problem, to enable programme planners to develop an appropriate strategy for combating TB in the country and to assess trends of the disease burden over time. The present study provides an estimate of the prevalence of bacteriologially positive pulmonary tuberculosis in Jabalpur district of Madhya Pradesh, central India.

Over the last five decades, a number of TB prevalence surveys have been carried out in different parts of the country by various agencies/researchers (Table 4) [2], [7]–[18]. The National sample survey conducted by ICMR during 1955–58 observed the prevalence of bacteriologically positive cases ranging between 200 to 800 per 100,000 with the average of 400/100,000 population [2]. Prevalence in the general population ranged from 144 per 100,000 in Wardha district, Maharashtra State to 1,090 per 100,000 in Raichur district, Karnataka [7]–[10]. There is a wide variation in TB disease prevalence amongst different tribal populations in the country ranging from 133 per 100,000 amongst the tribal population in Wardha district, Maharashtra to 1518 per 100,000 amongst the Saharia, a primitive tribe of MP [10]–[18]. However, it needs to be noted that many of these studies were conducted at different time periods using different methodologies and definitions.

**Table 4:** Prevalence of pulmonary tuberculosis in different parts of India.

Author & Year	Area/State	Population covered	Screening method	TB prevalence per 100,000	Reference
National sample survey, 1959	Various states of India	2,68,590	X-ray	200–800	[2]
National Tuberculosis Institute, Bangalore, 1974	Rural population of Banglore district, South India	65,647	X-ray	337–406	[7]
Gopi PG et al, 1997	Rural and urban population of Raichur district, South India	40,496	Symptom	1,090	[8]
Datta M et al, 2000	North Arcot Plains, Tamil Nadu, South India	64,077	Symptom & X-ray	800	[9]
Narang P et al, 1999	Rural population in Wardha district, Maharashtra	96,425	Symptom	144	[10]
Narang P et al, 1999	Tribal population in Wardha district, Maharashtra	17,841	Symptom	133	[10]
Datta M et al, 2001	Tribal population of Jawadhu hills, North Arcot District, Tamil Nadu, South India	26,320	Symptom & X-ray	840	[11]
Mayurnath S et al, 1984	Pahadis (Kashmiri tribals), Kashmir valley	14,154	X-ray	260	[12]
Chakma T et al, 1996	Saharia primitive tribe, Sheopur district, Madhya Pradesh	11,097	Symptom	1,270	[13]
Murhekar MV et al, 2004	Tribal population of Car Nicobar, A& N islands	10,570	Symptom	740	[14]
Bhat Jyothi et al, 2009	Tribal population of Madhya Pradesh, Central India	22,270	Symptom	387	[15]
Rao VG et al, 2010	Saharia primitive tribal community, Madhya Pradesh, Central India	11,116	Symptom	1518	[16]
Rao VG et al, 2010	Bharia primitive tribal community, Madhya Pradesh, Central India	2,586	Symptom	432	[17]
Yadav R et al, 2010	Baiga primitive tribal community, Madhya Pradesh, Central India	2,359	Symptom	146	[18]

The estimated prevalence of bacteriologically positive TB (smear and/or culture) in the present study is 255.3 per 100,000 population, which indicate that the TB situation in Jabalpur district is fairly comparable to the situation at national level with an estimate of 249 per 100,000 for India [19]. The finding that the prevalence increased with age and is higher in males than in females is consistent with the findings of other studies [4], [7], [9], [15]–[16], [20]–[22]. The present study shows significantly higher prevalence in rural areas as compared to the urban. The higher prevalence in rural area (469 vs 307 per 100,000) has also been reported by National Family Health Survey (NFHS-3) [22]. This could be due to poor performance of anti-tuberculosis services in rural areas due to various reasons such as a lack of awareness of the disease and of available services, difficult terrain resulting in irregular drug supply and poor supervision by programme officials. A Nationwide TB Prevalence Survey in the Philippines however found similar prevalence in urban and rural areas [23].

In the present study, the prevalence of smear positive PTB was 171.9 per 100,000 population and culture positive PTB 207.1, with a combined prevalence of bacteriologically positive PTB of 255.3. The prevalence of culture positive PTB was more than smear positive PTB in both rural and urban area, and also in both the sexes. These findings are comparable to other studies [8], [10], [11], [23]. Of the total 221 cases, 73 were positive by culture alone (Table 3). On the other hand, 40 cases were positive by smear but negative on culture. It is noted that 14 of these cases had a history of previous anti-TB treatment, 4 cultures were contaminated and 10 cases were reported as scanty i.e paucibacillary, smear positive. These factors might have contributed to the finding of such smear positive, culture negative cases. Similar findings have also been reported by other workers [8], [9], [10], [11], [15], [24]. However the findings highlight that without the availability of more sensitive diagnostic tools than microscopy alone, a significant proportion of infectious PTB cases in the community will be missed.

Although the RNTCP was piloted in 1993, it was only implemented in the study district during 2003–04. When effectively implemented over a number of years, the RNTCP has been shown to result in a significant decrease in the prevalence of TB disease, as demonstrated by TRC (NIRT) studies in Thiruvallur district in south India [25]. The performance of RNTCP in Madhya Pradesh state has been gradually improving since it's implementation, with an increase in the case detection rate from 42% in 2001 to 74% in 2010 and in the new smear positive cure rate from 83% in 2001 to 85% in 2009 [26], [27]. However, we cannot afford to be complacent, especially in view of the two-thirds of bacteriologically positive PTB cases that were detected in the survey and who were not on treatment i.e. for every one confirmed case on treatment there were two undetected infectious PTB cases in the community. These undetected untreated cases will continue to transmit the infection in the community. There is therefore an urgent need for strengthening case detection activities at the community level.

The limitations of the present study need to be considered while interpreting the results. The findings are based on the symptom elicitation alone. Chest X-ray was not used in this survey on account of the high cost of mobile X-ray unit, difficult terrain and particularly the unavailability of sufficient numbers of X-ray machines for the survey. A study in south India on the yield of cases by different screening methods [5] showed that symptom screening picked up about two-third of the cases, whereas Chest X-ray alone picked up more than three-fourth of the cases. With either method, the prevalence was underestimated by one-third in the former method and about one-fifth in the latter method. A correction factor (CF) of 1.7 was estimated for calculating total prevalence when symptom enquiry alone is used. Using this CF, the prevalence of tuberculosis in the present study would be estimated to be 434.0/100,000 population. Considering this, it appears that substantial numbers of additional cases would have been detected if chest X-ray screening had been included in the study methodology. Similar findings were also reported by other workers from tuberculosis surveys in different population groups [4], [11]. In addition, there was no information relating to health seeking behavior of the identified symptomatic individuals, and on the prevalence of childhood TB and HIV in this area. In spite of these limitations, the findings throw light on the current TB situation in this area of central India which will be useful in evaluating the impact of disease control measures and epidemiological trends by undertaking follow-up surveys in the coming years. Trends in symptom prevalence, prevalence of bacteriologically positive PTB in different age groups, age and sex-wise proportion of cases, case detection and cure rates will provide valuable information to programme managers regarding the impact of control measures at community level. The proportion of cases on anti-TB treatment out of the total number of detected cases during follow-up survey would further add to the seeing the impact of the control programme in the area.

## Conclusion

The study results provide vital information on the TB disease situation in Jabalpur district of the central Indian state of Madhya Pradesh, central India, and can serve as baseline data for future evaluation of the impact of disease control measures and epidemiological trends. The study findings suggest that the TB situation in this part of the country is comparable to that estimated for the national level. There is however, a need to maintain and further strengthen TB control measures on a sustained and long term basis in the area to have a significant impact on the disease prevalence in the community.
